# Fusion of a Variable Baseline System and a Range Finder

**DOI:** 10.3390/s120100278

**Published:** 2011-12-28

**Authors:** Javier Hernández-Aceituno, Leopoldo Acosta, Rafael Arnay

**Affiliations:** Department of Systems Engineering and Automatics, University of La Laguna, Avda. Francisco Sánchez s/n, 38204 La Laguna, Canary Islands, Spain; E-Mails: leo@isaatc.ull.es (L.A.); rafa@isaatc.ull.es (R.A.)

**Keywords:** correspondence problem, multiple baseline, variable baseline, pinhole camera, range finder, stereo vision, sensor fusion

## Abstract

One of the greatest difficulties in stereo vision is the appearance of ambiguities when matching similar points from different images. In this article we analyze the effectiveness of using a fusion of multiple baselines and a range finder from a theoretical point of view, focusing on the results of using both prismatic and rotational articulations for baseline generation, and offer a practical case to prove its efficiency on an autonomous vehicle.

## 1. Introduction

In this article we analyze, from an analytical point of view, the possibilities and limitations of the fusion of using multiple baselines and a range finder.

**Figure 1.**
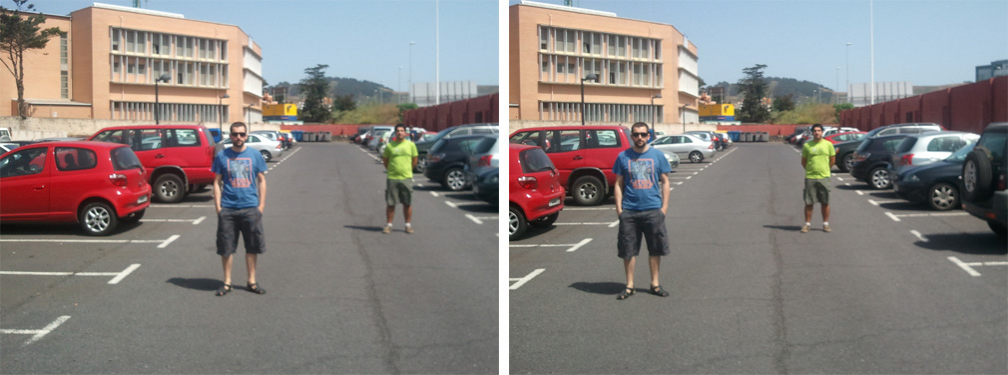
Example of images for stereo vision with ambiguities: the pedestrians must be correctly matched between the images or else their calculated depth will be wrong.

One of the most useful techniques to rebuild three-dimensional scenes from two-dimensional images is stereo vision, which uses the horizontal disparity between corresponding points in different images to calculate their depth position. The process of matching which objects from each image correspond to one another is a very complex process, especially if the analyzed scene contains repeated objects. For instance, Figure 1 shows an example of an image pair where even applying the epipolar restriction,...,*i.e.*, corresponding points must appear at equal vertical position,...,multiple ambiguities are bound to occur.

One of the main techniques to solve these ambiguities is the usage of multiple baselines, which provides the reconstruction process with more than two images of the same scene to compare. This tool has been studied before for different camera configurations and applying several processing algorithms, such as SSSD (sum of sum of squared differences) in inverse distance [1], adaptive windows [2,3], heuristics [4], focus/defocus support [5], or resolution adaptation [6], amongst others. Its practical efficiency has been tested and proved in experimental situations [7,8]; ultimately all research necessarily has to deal with the point correspondence problem, for which no analytical definition has been provided yet.

We intend to create a set of theoretical guidelines and restrictions to aid the design and construction of multiple and variable baseline systems, by accurately describing the relation between the usual configuration parameters and the matching process difficulty. Our research follows a strictly numerical approach, assuming ideal pinhole cameras with exact precision, and for two particular configurations: a classic linear baseline for two cameras at a variable distance, and a pair at a fixed distance with a variable orientation.

A laser range finder is then included in the vision system to enhance the results, greatly improving their precision for a large set of configurations. This device has been largely studied and customized in the past, by the addition of dynamic range enhancement [9] or amplitude modulation [10] among others, and used for many different tasks, such as map construction [11], landmark detection [12], ampacity measurement [13], inertial navigation [14], and space exploration [15,16]. TOF (Time of Flight) cameras [17–19] have also been used in sensor fusion with camera baselines, usually producing good results.

A multiple baseline system is bound to produce ambiguous results, in form of sets of points that must be matched to provide the location of the corresponding physical objects. The fusion procedure consists of comparing the output data of a laser range finder device with that of the baseline calculations, so that only one of the possible interpretations of its results is proved right.

This work is part of a project for the development of a low-cost smart system. It is designed for passenger transportation and surveillance in non-structured environmental settings, that is the Technical and Renewable Energies Institute (ITER) facilities in Tenerife (Spain). This features a housing complex of twenty-five environmental-friendly dwellings. One of the key elements of this task is obstacle detection and scene understanding, so the first stage of this process is to determine potential obstacles’ locations and approximate shapes.

The base system used for this project was the VERDINO prototype, a modified EZ-GO TXT-2 golf cart. This fully electric two seat vehicle was to operate automatically, so it was equipped with computerized steering, braking and traction control systems. Its sensor system consists of a differential GPS, relying on two different GPS stations, one of them being a fixed ground station. Its position is defined very accurately, so it is used to estimate the error introduced by each satellite and to send the corrections to the roving GPS stations mounted on the vehicle. The prototype’s three-dimensional orientation is measured using an Inertial Measurement Unit (IMU). This unit features three accelerometers, three gyroscopes, three magnetometers, and a processor that calculates its Euler angles in real time, based on the information these sensors provide. An odometer is also available and serves as a back-up positioning system in the event of a GPS system failure.

The prototype also includes several Sick LMS221-30206 laser range finders and a front platform that supports a vision system, which consists of two conventional and thermal stereo cameras. This platform moves to adjust the cameras’ height and rotation, allowing them to see around curves and on irregular terrain. This vision system was built to detect obstacles and the unpainted edges of roads, and will serve as grounds for our current research.

## 2. Methods

In this section we analyze two forms of baseline generation—variation of length or rotation—and generalize the common aspects of both procedures. We also present their fusion with a range finder system, study the effect of cardinality differences among sets of point projections, and evaluate possible irresolvable configurations.

**Figure 2.**
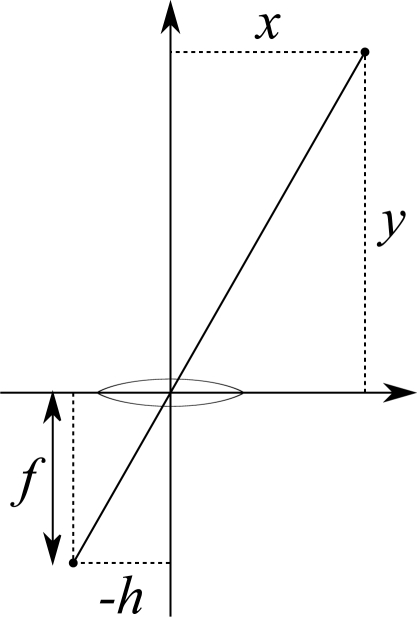
Parameters *f*, *h*, *x* and *y*.

Our nomenclature considers the cameras’ parameters are a constant focal distance *f* and, for each real point (*x, y, z*), a horizontal projection position *h* corresponding to *x; y* will be the depth value which must be calculated. The relation amongst these values is provided by Equation (1) and visually explained by Figure 2. The coordinate origin is located in the center of the camera lens, which would cause the focal distance to become negative; since we are only concerned about the parameters’ relation, we consider the projected image of point (*x, y*) to actually be (−*h*, −*f*), so our calculations are coherent.

(1) $$\frac{h}{f}=\frac{x}{y}$$

The distance between two hypothetical cameras 1 and 2 is named *b*_12_. For simplicity’s sake we will normalize all cameras’ *h* values to the same focal *f* value. Every point will produce one *h* on each camera; by combining them we can calculate its original position. Setting the coordinate origin at the tip of camera 1 we obtain Equation (2).

(2) $$\begin{array}{r} \frac{h_{1}}{f}=\frac{x_{12}}{y_{12}}\\ \frac{h_{2}}{f}=\frac{x_{12}-b_{12}}{y_{12}} \end{array}\}\;\left(x_{12},\;y_{12}\right)=\left(h_{1}\;\frac{b_{12}}{h_{1}-h_{2}},\;f\frac{b_{12}}{h_{1}-h_{2}}\right)$$

Ambiguities occur when similar pixels produce a set of more than one *h* value for each camera. Only one of their many possible combinations will be correct, and the rest will produce spurious points. We can obtain a third image by altering the baseline’s length or rotation—that is, using multiple baselines—therefore obtaining a new set of *h* values. Only one of the combinations of every two sets is bound to produce the correct positions, and it will necessarily coincide with that of any other pair of sets. This way we can determine the correct combination and the exact location of the analyzed points.

This spatial coincidence is defined as the Euclidean distance between the point sets calculated with the correct *h* set combination, which is expected to be zero. Since our research assumes calculation errors are to be expected in real measurements, we define a threshold value which equals the difference between the set distance for the correct *h* combination and the shortest for the wrong ones. We studied the optimal baseline configurations as those that make this threshold value greatest.

### 2.1. Length Variation

The first camera configuration lets camera 2 move towards or away from camera 1. The distance it moves from its original position will be named Δ*b*; we consider its final position as a new camera, so its *h* values will be named *h*_3_. Using cameras 1 and 3 we obtain Equation (3). When the choice of h values produces the correct points, the distance between points (*x*_12_, *y*_12_) and (*x*_13_, *y*_13_) is expected to be zero.

(3) $$\left(x_{13},\;y_{13}\right)=\left(h_{1}\;\frac{b_{12}+\Delta b}{h_{1}-h_{3}},\;f\frac{b_{12}+\Delta b}{h_{1}-h_{3}}\right)$$

We express the difference amongst *h* set combinations as the sum of the Euclidean distance between their calculated points; as explained before, the correct combination should result in a null distance. The distance between two points calculated with the same *h*_1_ value is calculated by Equation (4). Equation (4) will not provide a valid result whenever *h*_1_ equals *h*_2_ or *h*_3_. This can happen for two reasons.

(4) $$\begin{array}{ll} d\left(h_{1},\;h_{2},\;h_{3}\right) & =\sqrt{{\left(x_{12}-x_{13}\right)}^{2}+{\left(y_{12}-y_{13}\right)}^{2}}\\ & =\sqrt{\left(h_{1}^{2}+f^{2}\right){\left(\frac{b_{12}}{h_{1}-h_{2}}-\frac{b_{12}+\Delta b}{h_{1}-h_{3}}\right)}^{2}}\\ & =\left|\frac{\sqrt{h_{1}^{2}+f^{2}}}{h_{1}-h_{3}}\left(\frac{h_{2}-h_{3}}{h_{1}-h_{2}}\;b_{12}-\Delta b\right)\right| \end{array}$$

- If the distance between camera 1 and either of the rest is zero (*b*_12_ = 0 or Δ*b* = −*b*_12_) both would provide the same information. In this case said camera is redundant and its data will not contribute to the result.
- Otherwise the *h* values will coincide because the light beams they represent are parallel. If that is the case, any distance related to the point resulting from their crossing (and therefore that of the set it belongs to) is infinite, which would discard it as non valid.

Further study of this case showed an important detail. Let us consider two hypothetical points, *P_a_* and *P_b_*, which produce this effect for cameras 1 and 2—that is, their horizontal projections will be *h*_1*a*_ and *h*_1*b*_ for the former and *h*_2*a*_ and *h*_2*b*_ for the latter; we will assume *h*_1*a*_ = *h*_2*b*_. Resorting to Equation (2), we obtain two points for each combination of *h*.

(5) $$\begin{array}{cc} \begin{array}{l} P_{a}=\left(h_{1a}\;\frac{b_{12}}{h_{1a}-h_{2a}},\;f\frac{b_{12}}{h_{1a}-h_{2a}}\right)\\ P_{b}=\left(h_{1b}\;\frac{b_{12}}{h_{1b}-h_{2b}},\;f\frac{b_{12}}{h_{1b}-h_{2b}}\right) \end{array}\} & \begin{array}{l} Q_{a}=\left(h_{1b}\;\frac{b_{12}}{h_{1b}-h_{2a}},\;f\frac{b_{12}}{h_{1b}-h_{2a}}\right)\\ Q_{b}=\left(h_{1a}\;\frac{b_{12}}{h_{1a}-h_{2b}},\;f\frac{b_{12}}{h_{1a}-h_{2b}}\right) \end{array}\} \end{array}$$

Since they are different combinations, we know the *P* points and the *Q* points will not exist at once. However, point *Q_b_* cannot be calculated because its denominators are null. This immediately discards all the points that belong to its combination of *h* values (namely *Q_a_*) and, as there are no other available combinations, proves *P_a_* and *P_b_* as the real points.

Let us now suppose the light beams are not parallel, but divergent; this would mean that *h*_1*a*_ > *h*_2*b*_ if *b*_12_ > 0 and *h*_1*a*_ < *h*_2*b*_ if *b*_12_ < 0. In either case, the *y* coordinate of point *Q_b_* would be negative, which is impossible since the point would have to be behind camera 1. Again, since this particular point is discarded, the whole combination is rejected.

If more than two points are being considered, the rejection of one of them would immediately invalidate its whole set. This must be taken into account, since when a particular combination is discarded for two cameras, adding a new baseline would be unnecessary and would only add useless data. Therefore, since the light beams are more likely to be divergent the closer the cameras are, for ideal pinhole cameras ambiguity can be solved by making baselines shorter.

### 2.2. Baseline Rotation

The second camera configuration sets the two cameras on the edges of a rotary rigid body. Its axis is placed exactly between the cameras, so that the rotation radius is half the baseline length: 2*r* = *b*_12_; the orientation angle is named *θ*.

Since cameras 1 and 2’s relative position remain equal to the previously studied case, their points’ location can still be calculated using Equation (2), whereas its corresponding image using 1 and 3 now corresponds to Equation (6a). Using this we can now recalculate the distance equation for two points, as seen in Equation (6b).

(6a) $$\left(\begin{array}{c} x_{13}\\ y_{13} \end{array}\right)=\left(\begin{array}{c} \frac{h_{1}\;b_{12}}{2}\;\frac{h_{3}\;\text{sin}\;\theta +f\;(1+\text{cos}\;\theta )}{\left(h_{1}\;h_{3}+f^{2}\right)\;\text{sin}\;\theta +\left(h_{1}-h_{3}\right)\;f\;\text{cos}\;\theta }\\ \frac{f\;b_{12}}{2}\frac{h_{3}\;\text{sin}\;\theta +f\;\left(1+\text{cos}\;\theta \right)}{\left(h_{1}\;h_{3}+f^{2}\right)\;\text{sin}\;\theta +\left(h_{1}-h_{3}\right)\;f\;\text{cos}\;\theta } \end{array}\right)$$

(6b) $$\begin{array}{ll} d\left(h_{1},\;h_{2},\;h_{3}\right) & =\sqrt{{\left(x_{12}-x_{13}\right)}^{2}+{\left(y_{12}-y_{13}\right)}^{2}}\\ & =b_{12}\;\sqrt{h_{1}^{2}+f^{2}}\;\left|\frac{1}{h_{2}-h_{1}}-\frac{h_{3}\;\text{sin}\;\theta +f\left(1+\text{cos}\;\theta \right)}{2\left(h_{1}\;h_{3}+f^{2}\right)\;\text{sin}\;\theta +2\left(h_{1}-h_{3}\right)\;f\;\text{cos}\;\theta }\right| \end{array}$$

Divergence conditions must also be taken into consideration, but the relation between *h*_1_ and *h*_3_ values is now more complex. It can be simplified by checking that *y*_13_ must be strictly greater than zero for all valid configurations.

### 2.3. Generalization

Our starting data will be one set of *h* values for each camera: *I* = {*h*_1_}, *J* = {*h*_2_} and *K* = {*h*_3_} for cameras 1, 2 and 3 correspondingly. We assume each *h* value will belong to only one real point; each *h* set combination can be understood as the direct matching between the values of set *I* and those of one permutation of *J* and *K*, which we named *J^P^* and *K^P^*. Applying this convention to Equation (4) and considering the distance between two sets as the sum of the distances between their corresponding points, this value is given by Equation (7), where function *d* is either Equation (4) or Equation (6b).

(7) $$D\left(I,\;J^{P},\;K^{P}\right)=\sum_{i=1}^{\left|I\right|}\;d\left(I_{i},\;J_{i}^{P},\;K_{i}^{P}\right)$$

This value will be close to zero when the combination of permutations results in the correct points set. We intend to calculate the optimal value of Δ*b* or *θ* such that the difference between the right combination and the wrong ones, given as their *D* value, is greatest. The minimum value for this difference is the threshold value.

### 2.4. Sensor Fusion

Most methods that calculate distance maps from conventional cameras are based on the usage of multiple baselines, such as [1] and [2]. However, these approaches are limited to the cameras’ inherent error, which greatly increases over object distance. The precision of a multiple baseline system can be improved by adding a range finder device, which has a much lower inherent error, as we experimentally prove in Section 4. Our system always considers the cameras as its main sensor system, to be complemented with the laser device. Therefore, if an obstacle is not observed by the camera, its range data is not used.

By placing the range finder device directly over camera 1, most non-infinite threshold configurations can be easily solved using the depth value of all visible points, not needing a third camera. Our fusion system deals exclusively with ambiguous point distributions, which are reduced subsets of the stereo output. The stereo pair will detect an ambiguity every time the calculations of the point locations produce multiple results; as explained in Section 2, this will occur when the perceived distance between any two points of the input set is shorter than the camera baseline.

The calculated point locations are then compared to the corresponding beams of the range finder, in order to find the most probable combination. Therefore, we do not need a generic sensor fusion system, such as traditional Kalman filters [20] or hardware-level fusion, but a simplified *ad hoc* system that solves ambiguities for small point sets. In order to correctly select the most likely matching between the output data of the range finder and the camera baseline, a proximity measurement was designed as follows.

Consider the range finder provides its information as a certain *Z* (*α*) function that returns the depth value for each vision angle *α*. This angle can be easily calculated for each *h*_1_ value, so its corresponding *h*_2_ value is solved using Equation (8). This way, the point positions can again be calculated using Equation (2).

(8) $$Z\left(\text{arctan}\;\frac{f}{h_{1}}\right)=\sqrt{x_{12}+y_{12}}=\sqrt{h_{1}^{2}+f^{2}}\left|\frac{d_{12}}{h_{1}-h_{2}}\right|$$

However, a real range finder system will not provide these values accurately, but limited to a certain number of angles. The point cloud it returns is processed into a set of centroids and radii that describe the approximate location and size of visible objects. Our particular case, a vision system for a vehicle prototype, deals with pedestrians and other highly vertical objects as obstacles, which allows the application of some theoretical simplification. The measurements derived from a single horizontal sweeping plane can be extrapolated for the whole visible surface.

This new sets of centroids and radii will be named *C* and *R* respectively. For any *I* = {*h*_1_} and *J* = {*h*_2_} sets several point groups can be calculated. Their proximity to *C* weighted by *R* serves as a scale to determine the most acceptable group. For a single point given by any (*h*_1_, *h*_2_) couple and a particular centroid/radius pair, this proximity can be calculated as Equation (9).

(9) $$p\left(h_{1},\;h_{2},\;C,\;R\right)=\frac{R}{\sqrt{{\left(C_{X}-\frac{h_{1}\;b_{12}}{h_{1}-h_{2}}\right)}^{2}+{\left(C_{Y}-\frac{fb_{12}}{h_{1}-h_{2}}\right)}^{2}}}$$

The relation between a point and a centroid is not univocal, since they are not necessarily defined by *h* values. Therefore for each point group all possible combinations must be considered and all repetitions evaluated. A generalization for larger groups is then given by Equation (10). The threshold value can be redefined as the difference between the proximity value for the right point group and any other *h*-centroid combination.

(10) $$P\left(I,\;J^{P},\;C,\;R\right)=\sum_{i=1}^{\left|I\right|}\;\overset{\left|C\right|}{\underset{j=1}{\text{max}}}\;\left\{p\left(I_{i},\;J_{i}^{P},\;C_{j},\;R_{j}\right)\right\}$$

### 2.5. Effect of Cardinality Differences

Certain restrictions regarding sets *I*, *J* and *K* must be considered when calculating the threshold value for two or more points. If the number of elements in any of the sets is 1, all the points seen from its camera are aligned and, as only one combination will be possible, the solution is immediate using any of the other sets.

If all the sets’ cardinalities are different and greater than one there will be hidden points. The only way to solve this situation is to augment the smaller sets by repeating some of their elements, so that they all have the same cardinality. All possible combinations will have to be tested and their permutations generated so the problem can be solved normally.

Although this process would apparently increase the problem’s complexity, the number of permutations that must be considered may actually decrease for augmented sets. A set containing *n* elements would usually have *n*! possible permutations; if it is built out of only *m* < *n* different elements, there will be *m^n−m^* possible augmentations, since every element must appear at least once not to discard any valid *h* value. However, many of these permutations will be superfluous, since there will be repeated elements. The number of permutations of a size *n* sequence of *m* < *n* elements is given by Equation (11), where *a*, *b*, *c*, ... are the number of occurrences of each element (*a* + *b* + *c* + · · · = *n*).

(11) $$PR_{n}^{a,b,c,\cdots }=\frac{n!}{a!b!c!\cdots }$$

For our calculations, the maximum number of valid permutations for all the possible augmentations of a size *n* set with *m* different elements will be named *Q* (*m, n*), and the number of occurrences of *k^th^* element will be *i_k_*, 1 ≤ *k* ≤ *m. Q* will be calculated as the sum of all the valid permutations of each possible augmented set.

Each time an element reappears it will steal available spaces from the following, leaving at least one for each. Therefore, in each augmentation element *k* may appear from once to *M_k_* times, as seen in Equation (12a), except element *m* which must appear exactly *M_m_* times to fill in all remaining available spaces so the set has size *n. Q* is then given by Equation (12b); if repeated permutations were ignored, their number *T* would be Equation (12c).

(12a) $$M_{k}=n-\sum_{j=1}^{k-1}\;i_{j}-\left(m-k\right)$$

(12b) $$Q\left(m,\;n\right)=\sum_{i_{1}=1}^{M_{1}}\;\sum_{i_{2}=1}^{M_{2}}\cdots \sum_{i_{m-1}=1}^{M_{m-1}}\;PR_{m}^{i_{1},i_{2},\cdots ,i_{m-1},M_{m}}$$

(12c) $$T\left(m,\;n\right)=m^{n-m}n!$$

**Figure 3.**
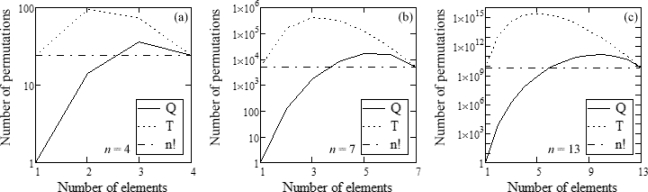
Number of possible permutations for variations of both the size desired for the sets (*n*) and the number of available elements (*m*).

Figure 3 shows the number of permutations, calculated both discarding repetitions (*Q*) and accepting them (*T*), and compared to the usual number of permutations (*n*!). As can be seen, avoiding repetitions greatly decreases the complexity of the problem for small values of *m*, and always provides better results than accepting all permutations. The overall form of the graph remains for any *n* value.

The number of calculations can still be reduced if we consider some combinations of these permutations will still be redundant. For instance, combining set *abca* with *xwxz* will be the same as combining it with *zwxx*.

Consider *A* is the set with a greatest cardinality, and *B*_1_ and *B*_2_ the other two. We will try matching them by pairs, remembering one matching of *h* values can not happen twice, or else we would be assuming two non-coincident lines can cross more than once. Sets *B*_1_ and *B*_2_ will have had to be augmented to suit *A*’s cardinality, which implies they will both have repeated elements which we must avoid combining incorrectly. Therefore and for simplicity’s sake we will start by combining set *A* with any of the *B* ones.

Since *A* has no repeated elements, its combination with every permutation of *B*_1_ will be valid; this makes *Q* (|*B*_1_|, |*A*|) valid combinations. Consider a new set *C* representing any of these new combinations. *C* cannot have repeated values either, since it necessarily contains one of *A*’s in each of its own. We may then repeat the process with *C* and *B*_2_, obtaining *Q* (|*B*2|, |*C*|) new combinations. Since there are *Q* (|*B*_1_|, |*A*|) possible *C* sets, the total number of combinations is *Q* (|*B*_1_|, |*A*|)×*Q* (|*B*_2_|, |*A*|), which often will improve the normal case |*J*|! × |*K*|!.

### 2.6. Irresolvable Configurations

Sometimes three cameras may not be enough to distinguish real points from spurious ones, when one of either is hidden from them all, even including the range finder device. This may only be solved by modifying the cameras’ position once again or by adding a fourth one.

**Figure 4.**
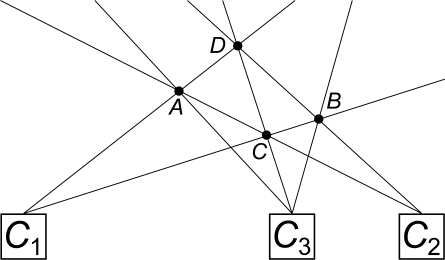
Irresolvable point-camera configuration.

For instance, Figure 4 shows an irresolvable point-camera configuration, where point *D* is occluded by *A*, *B* and *C* for cameras *C*_1_, *C*_2_ and *C*_3_ respectively. Since *C*_1_ can only see points *A* and *C*, and *C*_2_ sees *B* and *C*, using both at once *C* and *D* may be discarded as spurious; adding camera *C*_3_ will show *C* is real, but *D*’s existence can not be proved unless a fourth camera is used.

## 3. Results

### 3.1. Variable Baseline

Once the cameras have provided their *h* vectors for a particular set of points from a scene and the centroids/radii have been calculated from the range finder’s input, a computerized algorithm will have to compare all of their possible combinations. It will accept that with a smallest distance value 7, or complementarily that with a largest proximity value Equation (10), as generator for the position of the correct point set. Our intention is therefore to find which baseline variation parameters result in the most easily differentiable *h* sets, so that the chosen point set is more likely to be the correct one.

This differentiability is defined as the difference between the distance values for the correct point set and the closest wrong one. We studied this threshold value for all possible variations of both baseline length and rotation, along with the position and alignment of the original points. This way, the cameras can be arranged optimally depending on the location of the points, in order to improve the result of the matching algorithm. They are then compared with the values obtained by using proximities.

The studied arrangement included two points, (*x*_1_, *y*_1_) and (*x*_2_, *y*_2_), such that their middle point is (*x*, *y*), their distance is *δ* and the angle they form with the *x* axis is *α*—that is, Equation (13). The threshold value is studied for all of these parameters along with the camera baseline variations Δ*b* and *θ*.

(13a) $$\left(x_{1},\;y_{1}\right)=\left(x+\frac{\delta }{2}\;\text{cos}\;\alpha ,\;y+\frac{\delta }{2}\;\text{sin}\;\alpha \right)$$

(13b) $$\left(x_{2},\;y_{2}\right)=\left(x+\frac{\delta }{2}\;\text{cos}\;\left(\alpha +\pi \right),\;y+\frac{\delta }{2}\;\text{sin}\;\left(\alpha +\pi \right)\right)$$

**Figure 5.**
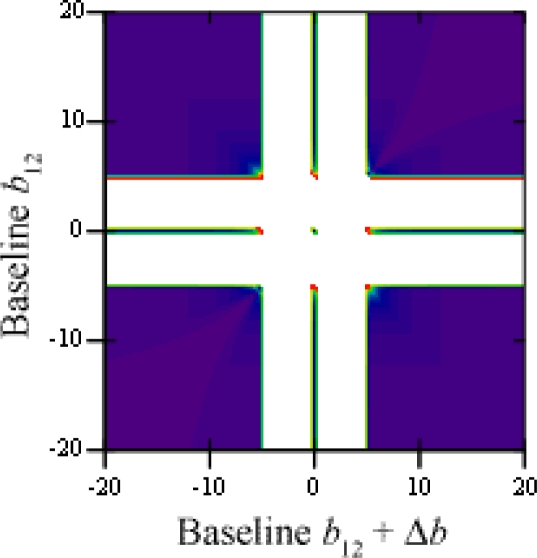
Threshold value for variations of baselines between cameras 1 and 2 and between 1 and 3.

The threshold value for variations of baselines between cameras 1 and 2 and between 1 and 3 is shown in Figure 5, using two points at equal depth (*α* = 0) and a gap of *δ* = 5 units; white areas are those where ambiguities do not occur. These happen whenever any of the baselines are shorter than the gap between points, that is |*b*_12_| < 5 and |*b*_12_ + Δ*b*| < 5, discarding *b*_12_ = 0 and *b*_12_ + Δ*b* = 0, which would make two cameras superimpose.

**Figure 6.**
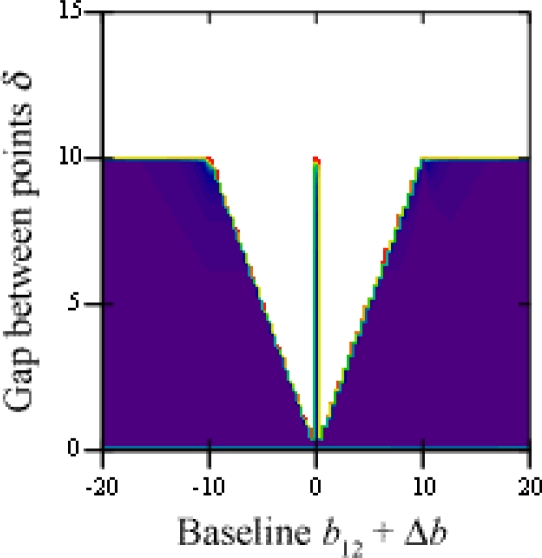
Threshold value for variations of the gap between points *δ* and the baseline between cameras 1 and 3.

In Figure 6, the threshold value is shown for variations of the gap between points *δ* and the baseline between cameras 1 and 3, the other remaining constant as *b*_12_ = 10. All ambiguities disappear whenever *δ* ≥ *b*_12_, regardless of the other camera’s position. Otherwise, the problem remains solved as long as the varying baseline is shorter than the gap between points, that is |*b*_12_ + Δ*b*| < *δ*.

**Figure 7.**
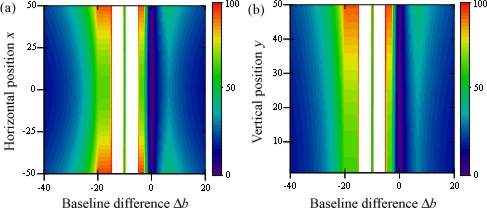
Threshold value for variations of *x* and *y* before Δ*b*.

**Figure 8.**
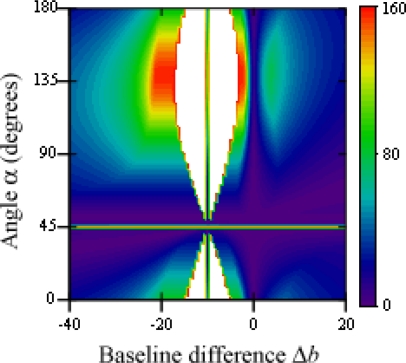
Threshold value for variations of rotation *α* and Δ*b*.

The threshold value for variations of *x* and *y* before the difference between baselines is shown in Figure 7 for *δ* = 5 and *b*_12_ = 10. Null values happen whenever two of the cameras superimpose (Δ*b* = 0 and Δ*b* = 10), and infinite values appear when the baseline is shorter than the gap between points (15 ≤ Δ*b* ≤ 5). For all other situations, the distance between the points and the cameras gradually improves the threshold value, making the correct solution easier to find. In Figure 8, the threshold value is shown for variations of rotation *α* and baseline difference. The angle the points form with the *x* axis greatly affects the threshold value, as it directly modifies the relative gap the cameras are able to see between the points. The case represented uses *x* = *y* = 50, and therefore a 45 degree angle makes both points appear aligned to camera 1. An angle 90 degrees greater or smaller (135 degrees in this case) makes the apparent gap, and therefore the threshold value, greatest.

**Figure 9.**
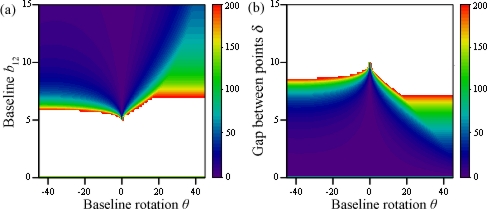
Threshold value for variations of *b*_12_ and *δ* before *θ*.

Figure 9 shows the threshold value for variations of baseline *b*_12_ and the gap between the studied points *δ* before the baseline rotation *θ*. The default values for these configurations are *x* = *y* = 50, *α* = 0, and, when needed, *δ* = 5 and *b*_12_ = 10. The former parameters are closely related since, as we studied for the previous case, anytime *b*_12_ is shorter than *δ* the problem’s solution is univocal. This is what causes the symmetry between graphs (a) and (b).

**Figure 10.**
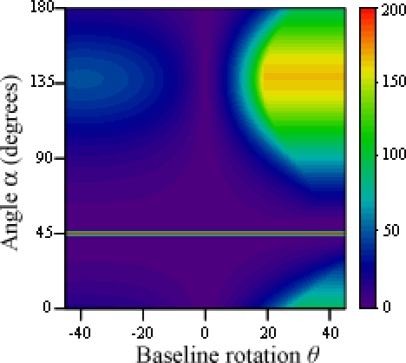
Threshold value for variations of *α* and *θ*.

The threshold value for variations of point rotation *α* and baseline rotation *θ* is shown in Figure 10. Again, since *x* = *y* = 50, a 45 degree angle makes both points appear aligned to camera 1, and an angle of 135 degrees makes the apparent gap greatest. This zone is however affected by the baseline rotation, as camera 3 provides a better image of the analyzed points when it faces them directly.

**Figure 11.**
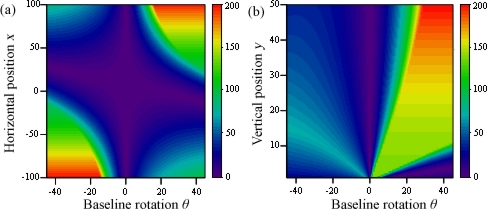
Threshold value for variations of *x* and *y* before *θ*.

In Figure 11, the threshold value is shown for variations of *x* and *y* before the baseline rotation, for *δ* = 5 y *b*_12_ = 10. Not only the results are better the further the points are from the cameras, but also when *θ* is such that camera 3 captures their image better. Graph (a) clearly shows that the sign of the rotation angle needs to be the same as the sign of *x*, otherwise the points will fall out of camera 3’s sight. Graph (b) however shows that this dependency to *x* is so great it completely alters the influence of parameter *y*: as before, it produces better results the greater it is, but only when *x* is located in a visible position.

As expected, whenever a baseline becomes shorter than the gap between the points—albeit greater than zero—the light beams become divergent. It was confirmed that variations of *x* and *y* did not affect the unambiguous areas. We also found that the threshold is proportional to the distance between the points and the cameras; this happens because as points move away from the cameras, the distance between spurious points also grows.

When studying the results of varying rotation *α*, we found that *x* and *y* also affect the result by modifying the relative gap the cameras perceive. If the points appeared aligned for any of the cameras, the problem was easily solvable as the *h* values for both points would be equal, but other close situations would only reduce the apparent gap and worsen the calculations results.

### 3.2. Range Finder

Complementing the described system, the range finder system can increase the number of situations in which a third camera is not necessary. We studied a new arrangement with two possible points, aligned to the same *h*_1_ value, such that only one of them may be real. A centroid is then swept over the visible area and the proximity based threshold value is analyzed. For those areas where the combined threshold is greatest, the problem can be solved using only two cameras and the range finder values.

**Figure 12.**
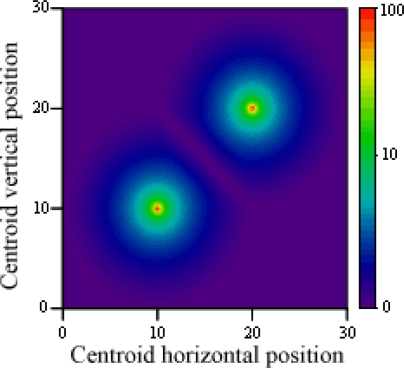
Proximity based threshold value for variations of the centroid position.

In Figure 12, the proximity based threshold value is shown for variations of the centroid position, for two incompatible points situated on (10, 10) and (20, 20), and a radius of 10 units. Whenever the distance between the centroid and a point is greater than its radius, the point is discarded; this way only a minimal intersection area is required to consider both points at once and decide which one provides a more feasible result, in combination with all other points from their groups.

Considering all calculations, an optimal combination of baseline and range finder can be designed. By placing this device over camera 1, all non-immediate point configurations can be first analyzed using their depth information, given as centroid/radius sets. Only when these data are not conclusive as means of scene reconstruction may the baseline parameters be modified.

Even though a rotary baseline might be easier to build than an extendable one, we have confirmed that the latter produces much better results than the former. Since our research requires one camera to remain static at all times in order to efficiently compare the calculated point sets, baseline rotation becomes inadequate. This is so because the third camera position is generally unable to produce infinite threshold situations—that is, whenever only two cameras are needed to find the point locations.

On the other hand, length variation avoids this problem so easily that a new possibility arises: according to our calculations and assuming ideal or precise enough cameras, a fixed 3-camera configuration could provide an optimal solution for all cases as long as two of them are as close possible. Most point configurations could be solved immediately by these alone.

## 4. Practical Case

We tested the presented algorithm in real life by placing two pedestrians in a partially controlled environment—namely the parking lot of La Laguna University’s Computer Engineering School—and using the VERDINO’s sensor system as input source.

**Figure 13.**
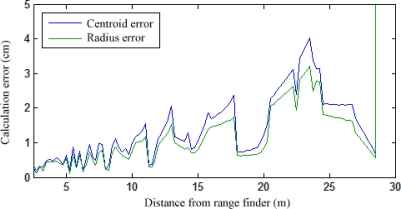
Range finder error between actual objects and calculated centroids and radii.

The range finder system consists of Sick LMS221-30206 laser models, which offer an angular resolution of 0.5°, a maximum range of 80 m, and systematic and statistic errors (at range 1–20 m) of ±35 mm and ±10 mm respectively. Figure 13 shows the experimental error of the device, as the average difference between the actual position and size of a pedestrian and its corresponding centroid and radius. As the distance between the device and an object increases, the number of laser beams which come in contact with it decreases, eventually making it impossible to estimate.

The stereo cameras are two Santachi DSP220x models, with a pixel resolution of 320 × 240, three color channels, an adjustable focal distance between 3.9 and 85.8 mm, and a lens angle of 47°. Their maximum precision at a distance of *d* meters is therefore 2.174 × *d* mm/pixel.

The data from the camera system recognize the obstacles, but they must be correctly matched to properly calculate their distance. By crossing this information with the range finder preprocessed output, spurious points were easily discarded as those that produced a minimum proximity value to the resulting set of centroids, as explained in Section 2.4.

**Figure 14.**
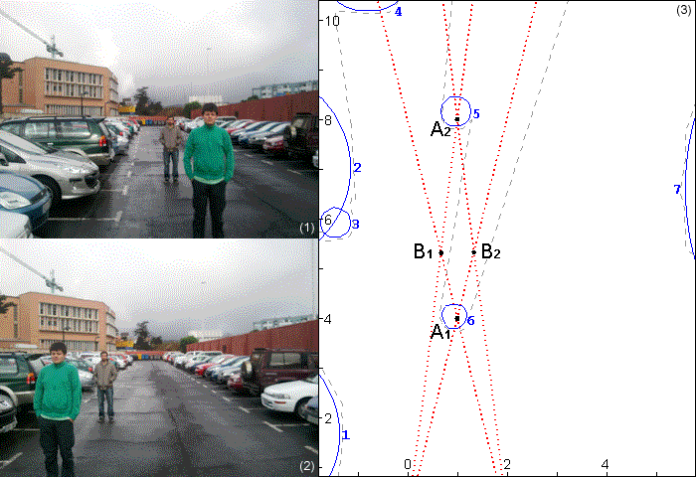
Input (1,2) and output (3) data of the sensor fusion algorithm. Units for (3) are given in meters and measured from camera 1.

The input and output data for a practical application of the sensor fusion algorithm are shown in Figure 14. The data from the cameras, represented as red lines, produces point pairs {*A*_1_, *A*_2_} and {*B*_1_, *B*_2_}, one of which must be spurious. The depth information given by the range finder is shown as a gray line, and the processed centroid/radius couples as blue circles. As can be seen, the wrong point set {*B*_1_, *B*_2_} does not match any centroids, therefore producing a much lower proximity value and being discarded.

In order to compare the performance of our sensor fusion system and a trinocular camera arrangement, both methods were tested in an environment with two pedestrians at various relative positions. These included variations of the pedestrians’ distance from the sensor system, horizontal position, separation, and rotation, similarly to our theoretical experiments in Section 3. The separation was always kept shorter than the baseline length, otherwise no ambiguities would occur.

**Figure 15.**
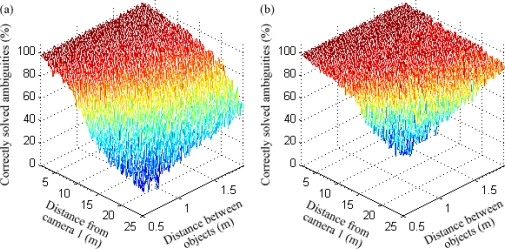
Experimental performance for variations of object distance and separation, for a trinocular system **(a)** and the stereo vision / range finder fusion system **(b)**.

**Figure 16.**
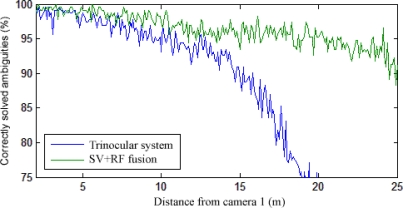
Comparison of experimental performance between a trinocular system and the stereo vision/range finder fusion system, for a fixed separation between objects.

The average percentage of correctly solved ambiguities is shown in Figure 15 for a variation in distance from the sensor system and separation. As expected, the results for both methods worsen when the objects are too close together. However, the fusion system always provides a much better performance than trinocular stereo vision, especially at large distances within the range of the range finder. A clearer comparison for a fixed distance of 1 m between the pedestrians is shown in Figure 16.

## 5. Conclusions

We have studied two different ways to obtain multiple baselines from a two-camera configuration, as well as the fusion of a single baseline with a range finder system, and analyzed their optimal settings to most accurately locate the observed points’ spatial positions. Our research has defined equations to efficiently evaluate solutions for the correspondence problem and proved which parameters provide the best results.

Considering ideal pin-hole cameras, a variable length baseline is most able to match points correctly the closer the cameras are to each other. A rotary baseline is more difficult to configure as its optimal orientation angle is directly related to the position of the analyzed points.

The performance of a stereo vision/range finder fusion system was tested and compared with a trinocular baseline arrangement. The experimental results proved that the fusion system generates a higher precision in a more extensive range.
